# Socioeconomic Inequalities in the Prevalence of Nine Established Cardiovascular Risk Factors in a Southern European Population

**DOI:** 10.1371/journal.pone.0037158

**Published:** 2012-05-29

**Authors:** Luís Alves, Ana Azevedo, Susana Silva, Henrique Barros

**Affiliations:** 1 Department of Clinical Epidemiology, Predictive Medicine and Public Health, University of Porto Medical School, Porto, Portugal; 2 Institute of Public Health - University of Porto (ISPUP), Porto, Portugal; 3 Santo André de Canidelo Health Family Unit, Vila Nova de Gaia, Portugal; Johns Hopkins Bloomberg School of Public Health, United States of America

## Abstract

The evaluation of the gender-specific prevalence of cardiovascular risk factors across socioeconomic position (SEP) categories may unravel mechanisms involved in the development of coronary heart disease. Using a sample of 1704 community dwellers of a Portuguese urban center aged 40 years or older, assessed in 1999–2003, we quantified the age-standardized prevalence of nine established cardiovascular risk factors (diabetes mellitus, hypertension, hypercholesterolemia, smoking, sedentariness, abdominal obesity, poor diet, excessive alcohol intake and depression) across SEP and gender categories. Data on individual education and occupation were collected by questionnaire and used to characterize SEP. The prevalence of seven out of nine well-established risk factors was higher in men. Among women, the prevalence of most of the studied risk factors was higher in lower SEP groups. The main exception was smoking, which increased with education and occupation levels. Among men, socioeconomic gradients were less clear, but lower SEP was associated with a higher prevalence of diabetes, excessive alcohol intake and depression in a graded mode. The historical cultural beliefs and practices captured throughout the lifecourse frame the wide socioeconomic gradients discernible in our study conducted in an unequal European developed population. While men were more exposed to most risk factors, the clearer associations between SEP and risk factors among women support that their adoption of particular healthy behaviors is more dependent on material and symbolic conditions. To fully address the issue of health inequalities, interventions within the health systems should be complemented with population-based policies specifically designed to reduce socioeconomic gradients.

## Introduction

Guidelines for coronary heart disease (CHD) prevention have increasingly considered the use of risk factor scoring systems [1], reflecting the importance of absolute individual risk [2]. More than 90% of incident myocardial infarctions are accounted for by diabetes, hypertension, hypercholesterolemia, smoking, sedentariness, abdominal obesity, poor diet, excessive alcohol intake and depression [3]. Furthermore, these factors tend to cluster [4], lending importance to the identification of and intervention on high risk groups.

Socioeconomic position (SEP) refers to the location groups of individuals hold within the structure of a society [5]. Education and occupation are proxy indicators of SEP that have been extensively used in social epidemiology. Educational attainment is generally acquired by early adulthood and is thought to capture the knowledge-related assets of an individual that contribute to the potential to modify behaviors and to promote healthier lifestyles [6]. Occupations indicate status and power that reflect the material and symbolic capital related to conditions at work [7]. Indicators based on occupations may be related to health not only through aspects such as differential accessibility to health care, but may also reflect social networks or psychosocial processes associated with health outcomes [8]. Poorer socioeconomic circumstances frequently lead to poorer health [9], although this general tendency sometimes hides important heterogeneity. Exceptions to this pattern may be an opportunity to understand how different dimensions of social stratification are differentially linked to health [10]. While education and occupation both reflect the position in the social and economic hierarchy held by individuals and families, their specific effects may suggest different mechanisms involved in the association between SEP and CHD.

Gender is another social dimension associated with health-related behaviors [11]. Males are more likely than females to engage in behaviors that increase the risk of disease [12]. Furthermore, evidence of gender heterogeneity in the relationship between SEP and CHD incidence has been reported, with stronger associations being found among women [13]. Thus, the evaluation of the gender-specific prevalence of cardiovascular risk factors across SEP categories can contribute to unravel mechanisms involved in the early development of CHD. Nevertheless, population-based studies comparing associations of multiple socioeconomic circumstances with a large set of important risk factors are scarce [14].

Portugal is recognized as one of Europe’s most unequal countries [15]. Furthermore, the strong austerity measures presently being adopted by the Portuguese government in response to the economical sovereign debt crisis are likely to further increase socioeconomic gradients in health. Given the large burden of disease associated with CHD in Portugal, it is important to assess whether inequalities are already present at the earliest stages of development of this condition. The identification of vulnerable societal groups will allow preventive efforts to be more efficiently channeled.

In this paper, we quantify the gender-specific prevalence of nine established risk factors for CHD across educational and occupational levels in a population-based sample of urban Portuguese adults.

## Methods

### Study Design and Sample Selection

The study design has been extensively described previously [16]. In brief, in 1999–2003, we assembled a representative sample of community dwellers of Porto, an urban center in the northwest of Portugal with almost 300,000 inhabitants at that time. We used random digit dialing of landline telephones to select households. The vast majority of houses (>95%) had a landline telephone at the time of this procedure. We used a table of random numbers to define the last four digits that are specific to individual houses, assuming the local prefix codes to limit the universe to the city of Porto. Non-existing numbers, those corresponding to fax numbers or telephone numbers of non-individual subscribers were ignored. The household was considered unreachable after at least four dialing attempts at different hours and including week and weekend days. Within each household, we selected a permanent resident aged 18 years or more using simple random sampling and refusals were not replaced. The proportion of participation was 70% [17] and the final sample size was 2485 individuals. The ethics committee of Hospital S. João approved the study. Participants provided written informed consent.

Subjects aged 40 years or older were eligible for the current analysis (n = 2000). Housewives who never had a paid occupation (n = 175) and those unemployed at the time of data collection (n = 67) were excluded. The Mini-Mental State Examination (MMSE) [18] was used to assess global cognitive function among participants aged 65 years and over. However, the predictive validity of this test may be educationally biased [19]. Therefore, we used the education-specific normative cut-off values of the MMSE for the Portuguese population [20]. Specifically, subjects were considered unable to provide reliable answers and excluded from the analysis when having a MMSE score below 16 if they had no formal schooling, 23 if they had up to 11 years of education, 28 if they had more than 11 years of education (n = 50). Subjects with missing information on education or occupation were also excluded (n = 4). Thus, the final sample comprised 1704 participants, 984 women and 720 men. Compared to participants, excluded subjects were more often women (women: 85.5% vs. 57.8%, p<0.001), significantly older [mean (standard deviation) age: 63.1 (11.6) vs. 57.6 (11.3), p<0.001], less educated [median (interquartile range) completed schooling years: 4 (4–8) vs. 6 (4–11.5), p<0.001] and with lower occupations (blue collar: 62.8% vs. 39.3%, p<0.001).

### Data Collection and Definition of Variables

Trained interviewers collected data on sociodemographic and behavioral characteristics, including diet, alcohol consumption, physical activity and smoking, and personal and family medical history, using structured questionnaires.

Age was categorized in 4 groups: 40–49, 50–59, 60–69 and 70 years or more. Education was recorded as completed years of schooling and aggregated in 3 categories: less than 5, 5–11, and more than 11 years. Occupations were classified by major professional groups, according to the National Classification of Occupations - version 1994 (NCO-94) [21] and grouped in three categories: upper white collar, lower white collar and blue collar. The upper white collar category comprised individuals classified in the upper three major groups of the NCO-94: executive civil servants, industrial directors and executives; professionals and scientists and middle management and technicians. The lower white collar category comprised individuals classified in the fourth and fifth major group of the NCO-94: administrative and related workers and service and sales workers. The blue collar category comprised individuals classified in the sixth to ninth major groups of the NCO-94. These major groups included farmers and skilled agricultural, fisheries workers, skilled workers, craftsmen and similar, machine operators and assembly workers and unskilled workers. Retired participants were classified considering their previous main occupation (n = 682). Similarly, housewives reporting a previous occupation were included in the analysis using this information (n = 80).

Participants were classified as never-smoker, former smoker (a person that stopped smoking at least 6 months ago) and current smoker, including both daily and occasional smokers [22]. For the present analysis, only current smoking was considered [3].

Physical activity was evaluated using a questionnaire exploring all professional, domestic and leisure time activities over the past 12 months [23]. Subjects reported their daily or weekly habits, detailing for each activity the average time spent in each group of activities such as rest (sleeping, lying awake), transport to and from work, work activities, household chores, sedentary leisure time activities and exercise. Each group of activities was assigned a metabolic equivalent (MET) value. One MET corresponded to an oxygen consumption of 3.5 ml/kg/min. These groups were categorized as very light, light, moderate and heavy activities, with average of 1.5, 2.5, 5.0 and 7.0 METs, respectively [24]. Energy expenditure was estimated by multiplying the related MET value times the self-reported duration of each activity, recorded in minutes per day. Participants were considered to be sedentary if they were classified in the lowest sex-specific third of a composite variable defined by the sum of daily leisure and sports energy expenditure. The cut-off values were 270 and 210 METs.min/day for men and women, respectively.

Dietary intake was estimated using an 82-item semiquantitative food frequency questionnaire, covering the previous year [25]. Each participant was asked about the mean frequency of consumption (nine categories ranging from “never or less than once a month” to “≥6 times a day”), the average portion consumed (lower, equal or higher than the mean portion size) and the seasonal variation of consumption. The questionnaire had 16 items related to vegetables and 16 items related to fruits. Only fresh fruits and natural fruit juices, and fresh vegetables and vegetable soups were considered. The food frequency questionnaire had been previously validated by comparison with four 7-day food records, each in a different season of the year [26]. A consumption of less than 5 portions of fruits or vegetables per day was considered low [27].

To estimate lifetime alcohol consumption, participants were asked about the lifetime mean frequency of consumption of different types of alcoholic beverages, including wine, beer, and spirits - liquors, gin, rum, vodka, cocktails or other mixed drinks. The period of highest exposure was considered. The average portion consumed was asked to be lower, equal or higher than a glass of 125 ml for wine, a bottle or can of 330 ml for beer, and a cup of 40 ml for spirits. The alcoholic beverages consumption was converted into total alcohol intake with the software Food Processor Plus® using an algorithm that assumed the following alcohol concentrations in volume: 12% for wine, 4.7% for beer, 25% for liquors and similar beverages, and 50% for vodka and the like. The algorithm was adapted to Portuguese drinks (e.g. Port wine). Two classes of alcohol consumption were defined by the cut points 15.0 grams per day (g/day) for women and 30.0 g/day for men, according to the American Heart Association recommendations [28].

The Beck Depression Inventory (BDI) [29] was used to quantify depressive symptoms. This scale includes 21 symptoms and attitudes, covering emotions, behavioral changes and somatic symptoms. Each item is rated on a 4-point intensity scale, with the overall score ranging from 0 to 63 and higher scores representing more severe symptoms. The questionnaire was self-administered at home. Subjects were given the questionnaire at the end of the interview with a prepaid envelope to return it with the responses. Illiterate subjects were not eligible to answer this self-administered questionnaire. For analysis, the BDI score was dichotomized using a cut-off of 15 points [30]. For the sake of simplicity, we will hereafter refer to BDI score above 15 as depression.

Anthropometrics were obtained after overnight fasting with the participant in light clothing and barefoot. Waist circumference was measured to the nearest centimeter using a flexible and non-distensible tape, midway between the lower limit of the rib cage and the iliac crest. Waist circumferences equal to or greater than 102 or 88 cm were used to define abdominal obesity in men and women, respectively [31].

Blood pressure was measured on a single occasion following the American Heart Association recommendations [32], with a standard mercury sphygmomanometer. Two blood pressure readings were taken with the participant resting for 10 minutes, and the mean of the two readings calculated. If the two readings differed more than 5 mm Hg, a third reading was taken and the mean of the two closest readings kept. Hypertension was defined as diastolic blood pressure ≥90 mmHg or systolic blood pressure ≥140 mmHg or being on antihypertensive drug treatment [33]. An overnight fasting blood sample was collected. Serum glucose level was determined using routine enzymatic methods and cholesterol and triglyceride levels were determined using standard enzymatic colorimetric methods [34], [35]. High density lipoprotein cholesterol levels were determined after precipitation of apolipoprotein B-containing lipoproteins [36]. Low density lipoprotein cholesterol (LDL-C) levels were calculated using the Friedewald equation [37]. Participants were considered diabetic if they had a fasting blood glucose measurement above 7.0 mmol/l [38], self-reported diabetes or were taking antidiabetic drugs. Hypercholesterolemia definition was based on the Third Report of the Expert Panel on Detection, Evaluation, and Treatment of High Blood Cholesterol in Adults [2]. An LDL-cholesterol cut-off value of 2.6 mmol/l was considered in subjects with self-reported personal history of acute myocardial infarction or angina pectoris. The same cut-off value was used if the participant was diabetic. For the remaining participants, the cut-off point was determined by a 2-step procedure. Firstly, the number of risk factors was counted: current cigarette smoking, hypertension, HDL-cholesterol <1.03 mmol/l, self-reported family history of premature acute myocardial infarction (acute myocardial infarction in male first degree relative <55 years or in female first degree relative <65 years), age ≥45 years in men or ≥55 years in women. For persons with 2 or more risk factors, assessment of 10-year risk of incident CHD was carried out using the Framingham risk prediction score to identify individuals whose risk warranted consideration of stricter cut-off values [39]. Specifically, if the 10-year risk of CHD event was >20%, a LDL-cholesterol cut-off value of 2.6 mmol/l was used; if the 10-year risk of CHD was ≤20%, a LDL-cholesterol cut-off value of 3.4 mmol/l was used. When less than 2 risk factors were present, a LDL-cholesterol cut-off value of 4.1 mmol/l was considered to define hypercholesterolemia. All participants on lipid lowering therapy were also considered to be hypercholesterolemic.

Participants with missing data in each outcome ranged from 0.82% (sedentariness and smoking) to 11.4% (hypercholesterolemia) of the total sample size. The exception was depressive symptoms that were quantified in 50.8% of participants for two main reasons. First, illiterate subjects were not eligible to answer this self-administered questionnaire. Second, the option of allowing participants to return the questionnaire later, after answering it at home, inevitably resulted in a lower proportion of participation specifically in this regard. Participants without information on depressive symptoms were older [mean (standard deviation) age: 58.7 (11.9) vs. 56.6 (10.7), p<0.001], less educated [median (interquartile range) completed schooling years: 4 (4–10) vs. 9 (4–12), p<0.001] and had occupations located at the bottom of socioeconomic hierarchy (blue collar: 47.9% vs. 31.1%, p<0.001). No significant difference was found in gender (women: 58.9% vs. 56.6%, p = 0.323).

### Statistical Analysis

Descriptive data are presented as count (percentage) for categorical variables. The χ^2^ test was used to compare proportions between groups. The t-test or Mann-Whitney-test was used to compare continuous variables between groups, as appropriate. The gender-specific crude risk factor prevalence was computed for each age category. The gender-specific risk factor prevalence in each education and occupation group was age-standardized using the European standard population. Ninety five percent confidence intervals were computed for each standardized prevalence estimate.

## Results

With the exception of abdominal obesity and depression, the prevalence of all other cardiovascular risk factors was higher in males than in females. In table 1, we present the gender-specific distribution of educational and occupational classes by age group. In both genders, younger participants had higher educational and occupational levels. In general, higher education was associated with higher occupation, although men with lower levels of education had upper white collar occupations more frequently than women (Table S1). In tables 2, 3, 4, we present the prevalence of risk factors across age, education and occupation categories.

**Table 1:** Distribution of educational and occupational classes by age category.

			Education (years)			Occupation		
			>11	5–11	<5	Upper white collar	Lower white collar	Blue collar
**Women**								
**Age (years)**								
40–49	n (%)		119 (38.1)	109 (34.9)	84 (26.9)	126 (40.4)	89 (28.5)	97 (31.1)
50–59	n (%)		82 (28.0)	69 (23.6)	142 (48.5)	100 (34.1)	75 (25.6)	118 (40.3)
60–69	n (%)		25 (11.0)	52 (22.9)	150 (66.1)	45 (19.8)	56 (24.7)	126 (55.5)
≥70	n (%)		19 (12.5)	27 (17.8)	106 (69.7)	26 (17.1)	28 (18.4)	98 (64.5)
	p^a)^			<0.001			<0.001	
**Men**								
**Age (years)**								
40–49	n (%)		80 (42.9)	74 (27.9)	40 (14.8)	100 (51.6)	49 (25.3)	45 (23.2)
50–59	n (%)		55 (29.6)	62 (24.9)	69 (24.8)	84 (45.2)	45 (24.2)	57 (30.7)
60–69	n (%)		24 (13.8)	68 (26.0)	96 (33.1)	66 (35.1)	46 (24.5)	76 (40.4)
≥70	n (%)		22 (13.8)	55 (21.2)	75 (27.5)	48 (31.6)	51 (33.6)	53 (34.9)
	p^a)^			<0.001			<0.001	

a) The χ2 test was used to compare proportions between groups.

**Table 2:** Prevalence of hypertension, diabetes mellitus and hypercholesterolemia across age, educationa) and occupationa) categories.

	Hypertension				Diabetes mellitus				Hypercholesterolemia			
	Women (n = 949)		Men (n = 688)		Women (n = 893)		Men (n = 662)		Women (n = 881)		Men (n = 629)	
	n/total	% (95%CI)	n/total	%(95%CI)	n/total	% (95%CI)	n/total	% (95%CI)	n/total	% (95%CI)	n/total	% (95%CI)
**Age (years)**												
40–49	83/289	28.7 (23.6–34.3)	65/181	35.9 (28.9–43.3)	12/289	4.15 (2.16–7.14)	8/177	4.52 (1.97–8.71)	79/287	27.5 (22.4–33.1)	92/175	51.6 (44.9–60.2)
50–59	151/289	52.2 (46.3–58.1)	101/181	55.8 (48.2–63.2)	16/267	5.99 (3.46–9.55)	21/176	11.9 (7.54–17.6)	121/265	45.7 (39.6–51.9)	108/168	64.3 (56.5–71.5)
60–69	168/221	76.0 (69.8–81.5)	139/180	77.2 (70.4–83.1)	34/206	16.5 (11.7–22.3)	26/172	15.1 (10.1–21.4)	135/205	65.8 (58.9–72.3)	118/164	72.0 (64.4–78.7)
≥70	134/150	89.3 (83.3–93.8)	112/146	76.7 (69.0–83.3)	21/131	16.0 (10.2–23.4)	16/137	11.7 (6.82–18.3)	76/124	61.3 (52.1–69.9)	71/122	58.2 (48.9–67.1)
**Education (years)** a)												
>11	93/234	50.1 (44.1–56.2)	84/168	56.0 (49.3–62.7)	6/229	2.38 (0.44–4.32)	10/168	6.81 (2.59–11.0)	83/230	41.5 (34.6–48.3)	99/159	65.2 (57.8–72.6)
5–11	113/245	49.5 (44.1–54.9)	151/248	56.8 (50.9–62.6)	20/239	8.89 (5.36–12.4)	22/237	8.93 (5.27–12.6)	94/230	42.4 (36.3–48.6)	131/226	55.8 (49.6–62.0)
<5	330/470	62.9 (58.2–67.5)	182/272	57.1 (50.6–63.7)	57/425	11.2 (8.30–14.2)	39/257	12.5 (8.57–16.4)	234/421	50.2 (45.1–55.2)	159/244	63.2 (56.1–70.2)
**Occupation** a)												
Upper white collar	118/283	49.0 (44.1–54.0)	160/282	56.0 (50.6–61.5)	12/275	4.64 (2.00–7.28)	27/271	9.58 (6.22–12.9)	107/275	43.7 (37.8–49.5)	166/255	65.9 (60.3–71.5)
Lower white collar	132/239	55.6 (50.0–61.3)	106/184	51.4 (44.1–58.6)	22/230	9.21 (5.64–12.8)	15/175	7.63 (3.68–11.6)	106/224	48.2 (41.8–54.5)	100/167	58.6 (51.0–66.1)
Blue colar	286/427	60.1 (55.7–64.6)	151/222	59.4 (52.6–66.2)	49/388	10.7 (7.82–13.5)	29/216	11.8 (7.45–16.1)	298/382	46.7 (41.8–51.7)	123/207	55.7 (48.5–62.8)

a) Prevalences were age-adjusted using the European standard population.

**Table 3:** Prevalence of abdominal obesity, sedentariness and smoking across age, educationa) and occupationa) categories.

	Abdominal obesity				Sedentariness				Smoking			
	Women (n = 969)		Men (n = 711)		Women (n = 975)		Men (n = 715)		Women (n = 974)		Men (n = 716)	
	n/total	% (95%CI)	n/total	%(95%CI)	n/total	%(95%CI)	n/total	% (95%CI)	n/total	% (95%CI)	n/total	% (95%CI)
**Age (years)**												
40–49	97/310	31.3 (26.2–36.8)	25/194	12.9 (8.52–18.4)	136/312	43.6(38.0–49.3)	87/194	44.8(37.7–52.1)	87/311	28.0(23.1–33.3)	90/194	46.4(39.2–53.7)
50–59	135/288	46.9 (41.0–52.8)	32/182	17.6 (12.3–23.9)	103/292	35.3(29.8–41.0)	69/186	37.1(30.1–44.5)	37/292	12.7(9.08–17.0)	56/186	30.1(23.6–37.2)
60–69	130/225	57.8 (51.0–64.3)	55/186	29.6 (23.1–36.7)	65/226	28.8(29.8–41.0)	56/187	29.9(23.5–37.1)	13/226	5.75(3.10–9.63)	44/187	23.5(17.6–30.3)
≥70	94/146	64.4 (56.0–72.1)	30/149	20.1 (14.0–27.5)	25/145	17.2(11.5–24.4)	31/148	20.9(14.7–28.4)	2/145	1.38(0.17–4.89)	19/149	12.6(7.86–19.2)
**Education (years)** a)												
>11	66/239	31.8 (25.8–37.8)	26/178	15.6 (10.0–21.3)	69/245	24.0(18.5–29.5)	58/181	32.1(25.5–38.8)	71/245	23.0(18.4–27.7)	60/181	28.7(22.7–34.6)
5–11	95/257	37.8 (31.8–43.8)	52/259	19.0 (14.2–23.6)	97/257	35.4 (29.9–40.9)	97/259	39.1(33.3–45.0)	43/256	15.3(11.2–19.4)	68/259	28.3(22.7–33.9)
<5	295/473	58.2 (53.3–63.2)	64/274	20.0 (14.8–25.2)	163/473	37.0(32.2–41.7)	88/275	36.0(29.3–42.6)	25/473	8.17(5.06–11.3)	81/276	36.0(29.4–42.7)
**Occupation** a)												
Upper white collar	83/291	31.0 (25.5–36.5)	53/294	17.5 (13.3–21.7)	77/297	24.0(19.2–28.9)	92/298	31.5(26.2–36.8)	71/297	20.0(16.1–24.0)	87/298	28.9(24.1–33.7)
Lower white collar	113/248	44.8 (38.8–50.8)	41/189	18.5 (13.1–23.8)	98/247	39.5(33.7–45.3)	63/190	34.0(27.0–40.9)	40/246	16.1(11.7–20.4)	58/190	34.1(26.8–41.3)
Blue colar	260/430	56.2 (51.4–61.1)	48/228	20.1 (14.4–25.7)	154/431	38.6(33.9–43.3)	88/227	42.7(35.9–49.5)	28/431	8.75(5.79–11.7)	64/228	32.6(26.1–39.2)

a) Prevalences were age-adjusted using the European standard population.

**Table 4:** Prevalence of low fruit and vegetable consumption, excessive alcohol intake and depression across age, educationa) and occupationa) categories.

	Less than 5 portions of fruits or vegetables per day				Excessive alcohol intake				Depression			
	Women (n = 970)		Men (n = 711)		Women (n = 947)		Men (n = 687)		Women (n = 490)		Men (n = 376)	
	n/total	%(95%CI)	n/total	%(95%CI)	n/total	% (95%CI)	n/total	% (95%CI)	n/total	% (95%CI)	n/total	% (95%CI)
**Age (years)**												
40–49	130/311	41.8 (36.3–47.5)	105/194	54.1 (46.8–61.3)	56/302	18.5(14.3–23.4)	91/181	50.3(42.8–57.8)	34/172	19.8(14.1–26.5)	6/99	6.06(2.26–12.7)
50–59	106/296	36.4 (30.9–42.2)	106/185	57.3 (49.8–64.5)	76/283	26.9(21.8–32.4)	116/179	64.8(57.3–71.8)	46/152	30.3(23.1–38.2)	12/105	11.4(6.05–19.1)
60–69	121/224	54.0 (47.2–60.7)	85/187	45.4 (38.2–52.9)	60/220	27.3(21.5–33.7)	118/183	64.5(57.1–71.4)	36/114	31.6(23.2–40.9)	7/103	6.80(2.78–13.5)
≥70	87/144	60.4 (51.9–68.5)	64/145	44.1 (35.9–52.6)	43/142	30.3(22.9–38.5)	80/144	55.6(47.0–63.8)	21/52	40.4(27.0–54.9)	12/69	17.4(9.32–28.4)
**Education (years)** a)												
>11	80/243	27.4 (22.3–32.5)	87/181	48.6 (41.4–55.7)	41/239	19.9(13.9–26.0)	78/174	45.0(37.6–52.4)	19/150	17.4(8.61–26.3)	8/112	6.50(2.10–10.9)
5–11	105/255	41.7 (35.4–47.9)	131/256	51.9 (45.8–58.0)	48/249	18.3(13.6–22.9)	137/248	53.3(47.2–59.5)	46/153	31.1(23.8–38.4)	13/140	9.34(4.44–14.2)
<5	259/472	53.5 (48.7–58.4)	142/274	57.6 (51.0–64.1)	146/459	30.1(25.5–34.6)	190/265	72.8(66.5–79.2)	72/187	35.1(28.0–42.2)	16/124	12.2(5.98–18.4)
**Occupation** a)												
Upper white collar	101/295	32.3 (27.0–37.6)	142/295	47.2 (41.7–52.6)	54/291	19.1(14.4–23.9)	144/289	49.8(44.1–55.6)	31/176	20.0(13.3–26.6)	14/172	7.37(3.67–11.1)
Lower white collar	115/245	48.3 (42.2–54.3)	102/189	54.7 (47.2–62.2)	47/240	19.4(14.4–24.5)	111/183	60.7(53.2–68.2)	39/141	27.4(20.1–34.6)	12/108	10.7(5.03–16.3)
Blue colar	228/430	50.8 (46.0–55.6)	116/227	54.2 (47.3–61.0)	134/416	30.3(25.8–34.9)	150/215	69.4(63.0–75.8)	67/173	36.7(29.6–43.8)	11/96	12.6(5.70–19.5)

a) Prevalences were age-adjusted using the European standard population.

### Education

Among women, we observed an age-independent variation in the prevalence of most cardiovascular risk factors across educational groups. Although in different directions, the largest relative differences were observed in the prevalence of diabetes mellitus and smoking. Specifically, the prevalence of diabetes mellitus was highest among women with less than 5 completed years of education (<5 years vs. >11 years: 11.2%, 95%CI: 8.30–14.2 vs. 2.38%, 95%CI: 0.44–4.32). Conversely, the least educated women smoked less frequently (<5 years vs. >11 years: 8.17%, 95%CI: 5.06–11.3 vs. 23.0%, 95%CI: 18.4–27.7). Abdominal obesity and consumption of less than 5 portions of fruits or vegetables per day were consistently more prevalent with decreasing levels of education. A prevalence gradient was not observed in sedentariness, depression, hypertension, hypercholesterolemia, excessive alcohol intake. Specifically, only the most educated women presented a lower relative frequency of sedentariness and depression. On the other hand, hypertension, hypercholesterolemia and excessive alcohol intake were more common in the least educated women.

Among men, there was a smaller variation in the relative frequency of most risk factors with education and gradients were less clear. The prevalence of diabetes mellitus, excessive alcohol intake and depression increased with decreasing levels of education. A prevalence gradient was not observed for hypercholesterolemia and fruit and vegetable daily consumption. Hypercholesterolemia was less prevalent among men with 5–11 years of completed education and low daily fruit and vegetable consumption was more prevalent among the least educated men. Hypertension, abdominal obesity, sedentariness, smoking and depression did not vary with education.

### Occupation

Among women, we observed a monotonic variation in the prevalence of the majority of risk factors across occupation categories. Although in different directions, the largest relative differences were also observed in the prevalence of diabetes and smoking. Whereas blue collar women were more commonly diabetic (blue collar vs. upper white collar: 10.7%, 95%CI: 7.82–13.5 vs. 4.64%, 95%CI: 2.00–7.28), smoking was less prevalent among women with less differentiated occupations (blue collar vs. upper white collar: 8.75%, 95%CI: 5.79–11.7 vs. 20.0%, 95%CI: 16.1–24.0). An inverse prevalence gradient was also observed for hypertension, abdominal obesity and depression, but not for sedentariness, fruit and vegetable consumption and alcohol consumption. Whereas sedentariness and low fruit and vegetable consumption were less frequent among upper white collar women, excessive alcohol intake was more common among blue collar women. Hypercholesterolemia was not associated with occupation among women.

Among men, we observed a gradient in the prevalence of excessive alcohol intake across occupational classes. Blue collar men presented a higher prevalence of excessive alcohol consumption (69.4%, 95%CI: 63.0–75.8) than men engaged in lower white collar (60.7%, 95%CI: 53.2–68.2) or upper white collar (49.8%, 95%CI: 44.1–55.6) occupations. Men engaged in lower occupations also tended to be more frequently depressed. Prevalence gradients were not observed for hypercholesterolemia, low fruit and vegetable consumption and sedentariness. Upper white collar men presented the highest prevalence of hypercholesterolemia and the lowest prevalence of low fruit and vegetable intake. Sedentariness was more prevalent only among male blue collar workers. Hypertension, diabetes mellitus, abdominal obesity and smoking were not associated with occupation.

## Discussion

As expected, the prevalence of seven out of nine well-established cardiovascular risk factors was higher in males than in females. Among women, the prevalence of most of the studied cardiovascular risk factors was higher in lower SEP groups. The main exception was smoking, which increased with education and occupation. Among men, lower SEP was associated with a higher prevalence of diabetes, excessive alcohol intake and depression in a graded mode.

Portugal is an interesting case-study due to the behavioral changes associated with a rapid transition to political democracy occurring in the seventies. This transition, catalyzed by the cultural elite, resulted in a rapid improvement in living standards, essentially marked by an increased access to consumer’s goods, not always accompanied by parallel social and cultural changes, particularly among the lowest SEP group. Although these changes were led to by an economic capital improvement across all social strata, this increment was proportionally smaller among the lower SEP groups, resulting in an increasingly unequal society in the economic dimension [40]. In fact, compared to other European Union nations, Portugal is still one of the most unequal countries [15]. The fact that our sample was composed by subjects aged 40 years or older around the year 2000 implies that participants have been differently exposed to both historic periods. We believe that the historical cultural beliefs and practices captured throughout the lifecourse frame the wide socioeconomic gaps discernible in our study.

While health related practices occurring in the early years of life impact on later health behaviors, they are also strongly associated with other social constructions such as education or occupation in adulthood. Consistent with other studies [41], [42], [43], [44], [45], socioeconomic gradients were steeper and more common among females. The clear gradients observed in women imply that the adoption of particular health behaviors is more dependent on material and symbolic conditions. For example, in our sample, the social meanings attached to smoking symbolize higher position for women, while fruits and vegetables consumption translate concern with the promotion of healthy lifestyles which is more evident among higher SEP women. The direction of these social gradients may seem contradictory, but they should be interpreted as reflecting the different mechanisms underlying the associations between SEP and specific risk factors, in a given secular time frame.

Hypertension was more prevalent among lower SEP women, as in other Mediterranean populations [46]. The fact that lower SEP women were more frequently aware of their condition suggests a differential healthcare service utilization pattern and its role in hypertension diagnosis. However, heterogeneity in the white coat effect across gender, SEP and behavior categories could also explain this observation. The ambulatory white coat effect has been reported to be higher in women, in older, obese and non-smoking subjects and in patients on antihypertensive drug treatment [47], [48]. If the same applies to our population, part of the increment in prevalence of hypertension among the lower SEP females may be explained by these factors, since hypertensive women were older, more obese, less frequently smokers and more frequently treated with antihypertensive drugs (data not shown).

Among women, education, but not occupation, was associated with hypercholesterolemia, which is consistent with other studies [49], [50]. Education has a more profound effect than occupation on behaviors and attitudes associated with hypercholesterolemia [51]. Hypercholesterolemia was less prevalent among the least educated women and among men with 5–11 complete years of education, which implies that education has a more expectable effect on hypercholesterolemia among females. Conversely, hypercholesterolemia was more prevalent among men engaged in upper white collar occupations. Although chance may account for this pattern, another explanation is possible. In Portugal, upper white collar occupations are more dependent on education in women than in men [52]. In our sample, among upper white collar subjects, the proportion of less than 5 completed years of education was 3 times higher in men than in women. Thus, the higher than expected prevalence of hypercholesterolemia in upper white collar men may be partially explained by the relative excess of less educated men in the highest occupations.

Smoking was more common among higher SEP women, reflecting Portugal’s position in the smoking epidemic [53]. Although most western populations are now at the fourth and last stage of the epidemic, with the prevalence declining in both sexes, and smoking being more common in lower social classes [14], southern European countries are still at the third stage, with the prevalence of smoking rapidly decreasing in men and reaching its peak among women [54], [55]. However, it is important to note that the data used throughout this paper were collected circa 2000 and do not represent the present Portuguese situation regarding smoking habits. In fact, according to the 2005/2006 National Health Survey [56], the most educated women presented a lower smoking prevalence than those with an intermediate educational level, reinforcing the expected dynamic nature of these associations. Furthermore, although our sample only comprised people 40 years or older, cohort effects were notable. The gradient between SEP and smoking was steeper among older women, when compared to those of younger age (data not shown). Although the cross-sectional nature of our study limits our ability to assess time-trends in health inequalities, we argue that this observation points to a decreasing impact of a higher social position on smoking among females.

The lowest levels of education or occupation were associated with a higher prevalence of sedentariness among women, which is consistent with other European populations [46], [57]. On the other hand, education was not associated with this outcome among men. Only blue collar men presented a higher prevalence of this risk factor. Either these men believe that their more physically demanding occupations argue against the need for any additional physical activity or they engage in less physically demanding leisure-time activities. These hypotheses are supported by the fact that occupational physical activity is an important determinant of leisure-time physical activity, particularly among individuals of low social standing [58].

The prevalence of excessive alcohol intake was higher and the socioeconomic gradient steeper in men than in women. In a review of social inequalities in alcohol consumption, the prevalence of heavy drinking tended to be higher among the most educated women and the least educated men. The exception to this pattern was observed in the Italian population, where heavy alcohol consumption was more common among the least educated women [59]. Our results agree with this observation and point to the specificity of southern European populations regarding this behavior.

Gender is grounded on cultural and ideological uses and meanings that vary with time and space [60]. In general, there is consensus within societies regarding what are adequate feminine or masculine characteristics [61]. Contrasting with femininity, hegemonic masculinity is commonly associated with potentially harmful health-related beliefs in contemporary western societies and men tend to experience comparatively greater social pressure to endorse corresponding behaviors [62]. High social classes can change their “gender’s repertoire” on health and illness narratives in order to maintain social distinction and authority, particularly in domains where conventional gender roles are threatened [63]. In endorsing hegemonic gender ideals with health behaviors, whereas men reproduce cultural beliefs that they are stronger and less vulnerable, women feel responsible for the promotion of healthy lifestyles [64]. The fact that most of our studied outcomes were more prevalent among men is in agreement with this vision, since most of these are related to unhealthy behaviors such as low consumption of fresh fruits and vegetables, smoking or drinking.

### Study Strengths and Limitations

This study adds to the literature a comparison of the gender-specific distribution of several important risk factors across categories of education and occupation in a European developed population. Specifically, the relevance of approaching this issue in Portugal is reinforced by reported large health inequalities [15]. The population-based nature of our study design allowed a comprehensive case ascertainment, minimizing detection bias. Most of the studied outcomes were defined using objective and valid instruments, avoiding problems of differential misclassification that are more common with the exclusive use of self-reported information. The studied population included subjects with prevalent coronary heart disease. We repeated the analysis after excluding 77 (7.8%) women and 65 (9%) men with personal history of prevalent coronary heart disease (self-reported angina or myocardial infarction, or electrocardiographic evidence of previous myocardial infarction). The standardized prevalence of all risk factors across education or occupation categories remained similar. Thus, the overall interpretation of results remains the same.

Some limitations of the present study warrant discussion. The proportion of participation of 70% may introduce selection bias. However, in a previous methodological article on the effects of the sampling procedures in this community-based study [17], Ramos et al. showed that participants were significantly younger and more likely to be females than non-participants, while there were no significant differences regarding education, occupation or marital status. Furthermore, non-participation had little or no impact on risk estimates of myocardial infarction, according to the demographic and social variables assessed, including education and occupation. This does not mean that data are free from selection bias, because it only involves the limited amount of information obtained from non-participants. Although the studied outcomes were not the same, the outcomes in the current study are in fact established risk factors for myocardial infarction. Therefore, non-response bias with respect to the characteristics assessed is unlikely to have played a major part in this study. Although limiting the generalizability of our findings, we excluded participants aged less than 40 years due to the low prevalence of most risk factors in younger ages and to avoid extreme cohort effects when comparing education or occupation categories. Also limiting the external validity of our study is the fact that our sample was exclusively drawn from an urban population. Non-inclusion of subjects belonging to rural settings may have led to an overall underestimation of the frequency of lower SEP subjects. However, we believe it is of interest to thoroughly characterize urban inequalities, especially since the proportion of urban population has been rising. Also, it is plausible that the same education and occupation might not represent the same SEP, and the frequency of cardiovascular risk factors by education/occupation may vary according to urban or rural settings. Women categorized as housewives are a heterogeneous socioeconomic group, and the absence of information on education or occupation of other household elements, usually the one with highest SEP, made it impossible to classify women that reported never having had a paid occupation. There is a large amount of studies showing that unemployed people incur in a multiplicity of elevated health risks, namely those related to cardiovascular disease [65], through a variety of mechanisms [66]. The small number of unemployed participants in our sample hampered our ability to analyze them as an independent stratum. Although we used a validated scale to quantify depressive symptoms, this measure is not equivalent to a clinical diagnosis of depression. Additionally, we could not assess this risk factor in a subsample of older, lower SEP subjects. Still, we observed an increase in depressive symptoms frequency across lowering categories of SEP, especially among women. Assuming subjects excluded from this analysis were more likely to be depressed, we may have underestimated the prevalence of this risk factor among subjects belonging to lower SEP groups.

### Conclusion

The historical cultural beliefs and practices captured throughout the lifecourse frame the wide socioeconomic gradients observable in our study. While men were more exposed to most risk factors, the clearer associations between SEP and risk factors among women support that their adoption of particular healthy behaviors is more dependent on material and symbolic conditions. Thus, the adoption of healthier lifestyles may depend on a reconfiguration of hegemonic gender roles. Although behavioral factors like smoking, physical activity and fruit and vegetable consumption account for an important fraction of cardiovascular disease, preventive efforts focusing entirely on individual behaviors are unlikely to significantly modify socioeconomic inequalities in health outcomes. To fully address the issue of health inequalities, interventions within the health systems should be complemented with population-based policies and health promotion initiatives specifically designed to reduce socioeconomic gradients.

## Supporting Information

Table S1 **Distribution of occupational classes by education category.**
(DOC)
