# Supplementary material for: Socioeconomic Inequalities in the Prevalence of Nine Established Cardiovascular Risk Factors in a Southern European Population
Source: PLoS One. 2012 May 29;7(5):e37158. doi: 10.1371/journal.pone.0037158 (PMC3362583; doi:10.1371/journal.pone.0037158)
Supplement: Table S1 — Distribution of occupational classes by education category. (DOC) [file pone.0037158.s001.doc]

Table S1 – Distribution of occupational classes by education category

|  |  | Women | | | Men | | |
| --- | --- | --- | --- | --- | --- | --- | --- |
|  |  | Upper white collar | Lower white collar | Blue collar | Upper white collar | Lower white collar | Blue collar |
| Education (years) |  |  |  |  |  |  |  |
| >11 | n(%) | 218 (88.9) | 25 (10.2) | 2 (0.8) | 162 (89.5) | 17 (9.4) | 2 (1.1) |
| 5-11 | n(%) | 73 (28.4) | 136 (52.9) | 48 (18.7) | 116 (44.8) | 93 (35.9) | 50 (19.3) |
| <5 | n(%) | 6 (1.24) | 87 (18.0) | 389 (80.7) | 20 (7.1) | 81 (28.9) | 179 (63.9) |
|  | p^a)^ |  | <0.001 |  |  | <0.001 |  |

a) The χ2 test was used to compare proportions between groups.
